# Effects of Water-Delivered Probiotics on Performance, Carcass Traits, Immunity, Blood Biochemistry, and Ileal Morphology of Broilers Reared at High Stocking Density Under Warm Ambient Temperature

**DOI:** 10.3390/ani16020328

**Published:** 2026-01-21

**Authors:** Ibrahim Al-Homidan, Abdulla Alsuqayhi, Osama Abou-Emera, Zarroug Ibrahim, Moataz Fathi

**Affiliations:** 1 Department of Animal and Poultry Production, College of Agriculture and Food, Qassim University, Buraydah 51452, Saudi Arabia; 2 Department of Poultry Breeding Research, Animal Production Research Institute, Agriculture Research Centre, Dokki, Giza 12618, Egypt; 3 Department of Medical Biosciences, College of Veterinary Medicine, Qassim University, Buraydah 51452, Saudi Arabia

**Keywords:** hot climate, immune response, probiotics, antioxidant profile

## Abstract

This study investigated the effects of dietary probiotic supplementation and stocking density on the growth performance, carcass traits, immunity, blood biochemical parameters, and ileal histomorphology of broiler chickens. A total of five hundred ten 1-day-old unsexed broiler chicks (Cobb 39) were allocated to three probiotic levels (0%, 0.1%, 0.2%) and two stocking densities (low vs. high). Results indicated that stocking density significantly influenced body weight from the third week onward, with birds reared under low density showing higher weight and better feed-to-gain ratio. Probiotic supplementation did not significantly affect weekly body weight, feed intake, or mortality, although mortality tended to be lower in probiotic-fed groups. Carcass traits and lymphoid organ indices were largely unaffected by treatments, except for a higher heart percentage in low-density birds. Cell-mediated immunity was enhanced under low stocking density, and probiotic supplementation at 0.2% increased the immune response at 48 h post-challenge. Blood biochemical analysis revealed significant effects of stocking density on total protein, globulin, and triglycerides, while probiotics reduced total lipid and LDL levels. Ileal histomorphology was significantly improved by probiotics, with increased villus height, crypt depth, and villus-to-crypt ratio. Similarly, low stocking density further enhanced these parameters. Overall, probiotic supplementation, particularly at 0.1%, combined with low stocking density, positively influenced gut morphology and immune responses, contributing to improved broiler health and performance.

## Simple Summary

Broiler chickens raised at high stocking density during hot weather often experience reduced growth and compromised health. Probiotics are increasingly used as alternatives to antibiotics to support gut function and immunity. This study evaluated two levels of Bacillus licheniformis administered through drinking water to broilers kept under low or high stocking density. Although probiotics did not significantly improve growth performance, they reduced mortality, improved blood lipid values, and substantially enhanced ileal villus morphology. Low stocking density consistently improved performance, immunity, and intestinal structure. These results highlight the potential benefits of water-administered probiotics and the importance of maintaining appropriate stocking densities under warm conditions.

## 1. Introduction

The broiler production sector is a promising industry in the Kingdom of Saudi Arabia, responsible for producing high quality of white meat. The birds are usually slaughtered at 26 to 32 days of age in fast production cycle. Many farmers resort to the irrational use of antibiotics to enhance the productive performance of their flocks. However, regulatory restrictions across the European Union, the United States, and many other countries are driving the development and adoption of natural additives as alternatives to antibiotics for promoting growth and preventing disease. There is no doubt that using probiotics in poultry feeding as a natural alternative can improve growth performance, intestinal health, and immunity, while also ensuring the safety of the meat. Probiotics as single- or multi-species can elicit different responses in birds [1,2]. Probiotics come in several forms for poultry use, including drops, sprays, granules, tablets, powders, and sachets [2].

Numerous studies have demonstrated the positive effects of probiotic administration on growth performance, feed efficiency, carcass yield, immune responses, and meat quality in chickens [3,4,5,6]. The effectiveness of probiotics can vary depending on factors such as type, strain, delivery route, dose, and duration of use [7]. Among probiotic bacteria, *Lactobacillus* and *Bacillus* are the most widely used in broiler production [4]. The positive effects of *Bacillus* spp. and *Lactobacillus* spp. probiotics in broilers have been documented previously [8,9,10]. Probiotic administration has been shown to significantly enhance gut health by regulating the gut microbiota, improving villus structure, and boosting immunity [11,12,13]. Additionally, their beneficial effects in alleviating the adverse impacts of heat stress on broilers have been reported [4]. Although increasing the number of birds per unit of space may appear more profitable, the negative effects of overstocking on health and performance can ultimately reduce economic returns and raise significant welfare concerns. The combined effects of high stocking density and elevated ambient temperature on broiler production have also been reported [14,15,16].

In experimental studies, probiotics can be administered through either feed or drinking water in broiler chickens, with feed being the most commonly used method [17,18]. At high temperatures, chickens increase their water intake and decrease their feed intake. Accordingly, administering probiotics via drinking water is advantageous, particularly for maintaining probiotic viability in heat-stressed broilers and ensuring uniform distribution. Another important benefit of adding probiotics to drinking water is the potential to support increased water intake while reducing feed consumption in birds under heat stress [19]. However, high stocking density and heat stress impair broiler performance and health, while probiotics delivered through drinking water may improve gut health and immunity. Therefore, exploring their combined effects could provide valuable insights. The objective of this study was to evaluate the potential effects of probiotic administration in alleviating the combined adverse effects of high stocking density and warm ambient temperature on growth performance, carcass quality, blood parameters, ileal histomorphology, and cell-mediated immunity in broiler chickens.

## 2. Materials and Methods

### 2.1. Study Site, and Ethical Statement

The experiment was conducted during May and June 2025 at the Poultry Research Farm of Qassim University, Saudi Arabia. All research procedures have been approved by the Committee of Research Ethics, Deanship of Graduate Studies and Scientific Research, Qassim University, Al-Qassim, Saudi Arabia (Approval # 25-32-01).

### 2.2. Experimental Design

The chicks used in this study were raised in a semi-closed house at the Poultry Research Unit of Qassim University during the summer season of 2024. The trial was conducted using a completely randomized design with a 3 × 2 factorial arrangement to examine the effect of probiotic administration (three levels) on broiler chicks reared under the stressful condition of high stocking density. The two stocking density rates included 12 birds/m^2^ as low stocking density (LSD) and 18 birds/m^2^ as high stocking density (HSD).

### 2.3. Chicks, Management, and Probiotic Supplementation

A total of five hundred ten 1-day-old unsexed broiler chicks (Cobb 39) were obtained from a commercial hatchery. All chicks had been immunized with the vaccines against Marek’s disease, Newcastle disease, and Infectious bursal disease at one-day old. The birds were reimmunized against Newcastle disease on the 12th and 24th day of age, and against Gumboro disease on the 14th and 23rd day of age. Prior to placement, chicks were weighed, divided into six subgroups and randomly distributed into 30 floor pens (replicates), with 5 replicates per subgroup. Each floor pen (110 cm wide × 180 cm long) was littered with wood shavings to a depth of 5–6 cm. Accordingly, each pen contained either 14 or 20 birds for the LSD and HSD groups, respectively. For probiotic supplementation, a substance containing 6.67 × 10^11^ colony-forming units (CFU)/g of *Bacillus licheniformis* (GalliPro-Tect-WS; Chr. Hansen, Milwaukee, WI, USA) was used at two levels. For low and high concentration levels of probiotics, 0.1 g and 0.2 g were dissolved in each liter of drinking water, respectively. The actual *Bacillus licheniformis* concentration was 6.67 × 10^9^ CFU/L and 13.35 × 10^9^ CFU/L for the low and high levels, respectively. Chicks that received fresh drinking water without supplementation served as the non-supplemented (control) group and were further distributed into low and high stocking density treatments. All experimental treatments were applied from the first day of age, and the trial was conducted continuously throughout the 35-day experimental period. Each pen was equipped with a pan feeder and two bell-shaped waterers. To ensure effective dissolution of the probiotics in the water and their delivery to the chicks in a fresh manner, the mixture was prepared and distributed daily for each replicate. The chicks were fed three different broiler diets (corn-soybean-based meal) until they reached 5 weeks of age (Table 1). The chicks had ad libitum access to the basal diet and tap water. Supplemental lighting was provided continuously. The broiler chicks were initially raised at a temperature of 32–34 °C for the first three days, followed by rearing at an ambient temperature of 30.4 ± 0.4 °C and 26.7 ± 0.3 °C for the high and low temperatures, respectively, until the end of the experiment.

### 2.4. Growth Performance

Body weight was measured weekly using an electronic balance. Feed intake was recorded from day 0 to day 35 on a replicate basis. Body weight gain (per pen) was calculated as the difference in weight from day 0 to day 35. Daily monitoring and recording on replicate basis were followed to determine the mortality during the experimental period. Feed intake was calculated weekly and adjusted for mortality as it occurred in each replicate. To calculate the feed conversion ratio (FCR), the feed intake for each replicate was divided by the total weight gain.

### 2.5. Carcass Yield, and Lymphoid Organs

To evaluate carcass characteristics, 10 chicks from each subgroup (sex balanced) were randomly selected at the end of the experiment and weighed. After a 12 h feed withdrawal, the birds were euthanized by jugular vein bleeding at a broiler slaughterhouse. After complete bleeding, the birds were defeathered. The feet, head, and wingtips were removed. Then the birds were opened and eviscerated to determine the dressed carcass weight. The whole leg, drumstick, thigh, breast muscles (major and minor) from the right side, and giblets (liver, heart, and gizzard) were dissected and weighed. Additionally, the lymphoid organs (thymus, spleen, and bursa of Fabricius) were excised, weighed, and recorded individually. All different parts of the carcass were expressed as a percentage of the live body weight.

### 2.6. Cellular Immunity Assay

The cutaneous basophil hypersensitivity test, a measure of T-cell-mediated response, was quantified using phytohaemagglutinin-P (PHA-P) via a toe-web injection technique. At the beginning of the 5th week of age, 60 chicks were randomly assigned (10 from each subgroup). Each bird was intradermally injected in the toe web with 100 μg of PHA-P (Sigma Chemical Co., St. Louis, MO, USA) in 0.1 mL of sterile saline. To quantify the inflammatory response, web thickness was measured using a constant-tension caliper before injection and at 24, 48, and 72 h post-injection. Web swelling was calculated as the difference between the thickness of the web before and after injection. The resulting swelling was interpreted as an index of cell-mediated immunocompetence.

### 2.7. Blood Biochemical Indexes

At the end of the experiment, blood was collected during slaughter from 10 birds per subgroup. The samples were collected in clean vials containing heparin as an anticoagulant. All blood samples were centrifuged at 1400× *g* for 10 min to separate the plasma for measuring blood metabolites. The plasma was then transferred into properly labeled vials and stored at −20° C for further analysis. Total protein, albumin, total cholesterol, triglycerides, total lipids, low-density lipoprotein (LDL), high-density lipoprotein (HDL), total antioxidant capacity (TAC), and Malondialdehyde (MDA) levels were determined using commercial kits (BioMérieux, Marcy-l’Étoile, France). Globulin was calculated as the difference between total protein and albumin.

### 2.8. Intestinal Histomorphological Measurements

At 35 days of age, the intestinal histomorphological measurements were determined in the same sacrificed birds. Immediately after slaughter, a 2 cm segment of the ileum (from the distal end of the lower ileum, 10 cm proximally to the ileocecal junction) was collected. These segments were flushed with phosphate-buffered saline (PBS, pH 7), fixed for 45 min in Clark fixative (Merck, Darmstadt, Germany) containing 25% glacial acetic acid and 75% ethanol, and then stored in 50% ethyl alcohol for longer preservation. For each segment, a 5 mm cross-section was cut using a microtome, placed on a glass slide, and stained with periodic acid–Schiff reagent for 2–3 min. Villus height and crypt depth were measured using a light microscope (Olympus CX31, Tokyo, Japan). Villus height was measured from the top of the villus to the top of the lamina propria, while the villus: crypt ratio was calculated as the ratio of villus height to crypt depth [19]. Three replicate measurements were taken for each variable studied from each sample, and the average values were used in the statistical analysis.

### 2.9. Statistical Analysis

Data were subjected to a two-way ANOVA using JMP software version 13.0 with probiotic level and stocking density as fixed effects, Institute, 2013 [20]. Sex was not included as a factor in the statistical model because the birds were raised in mixed-sex groups as in the commercial broiler production. Additionally, the studied traits were determined in a sex-balanced manner to ensure that any potential sex effect was distributed equally across all treatments. The statistical model is described as follows:

Y_ijk_ = μ + P_i_ + S_j_ + (PS)_ij_ + e_ijk_

where

Y_ijk_ = the observation taken on the kth individual,

μ = overall mean,

P_i_ = the fixed effect of the jth probiotic supplementation level,

S_j_ = the fixed effect of the ith stocking density,

(PS)_ij_ = interaction between probiotic supplementation level and stocking density,

e_ijk_ = random error assumed to be independent normally distributed with mean = 0 and variance = σ^2^.

Prior to statistical analysis, the data were examined for normality using the Shapiro–Wilk test, while the homogeneity was tested using Levene’s test. All results were presented as mean, and the variability in the data was expressed as pooled standard error of the mean. Chi-square analysis was applied for the mortality data. The significance of difference among the groups was assessed using Tukey post hoc analysis. Differences between means were considered significant at *p* < 0.05. Orthogonal polynomial contrast test for linear and quadratic effects was applied to describe the shape of the response to increasing concentrations of probiotic supplementation and to determine the best model fit. The responses in optimal parameters to the probiotic supplementation level can be modeled using the following quadratic equation:

Y = a + b_1_X_1_ + b_2_X_2_ + e

where Y = optimal response, a = intercept, bs = coefficients of the quadratic equation, Xs = probiotic levels, and e = error.

## 3. Results

### 3.1. Growth Performance and Mortality Rate

The effects of different levels of probiotics and stocking density on broiler growth performance are presented in Table 2. The initial weights of the birds were not significantly different, with an average body weight of 40.5 ± 0.14 g on the first day. Generally, probiotic supplementation did not affect body weight, weight gain, or feed intake. However, an increase in the feed-to-gain ratio was observed during the early weeks in birds receiving higher level of probiotics. This deterioration disappeared over the entire experimental period. In terms of mortality rate, the control group recorded the highest mortality (5.3%), while birds receiving probiotics at 0.1% and 0.2% had lower mortality rates (2.9% and 2.4%, respectively). At 3 weeks, birds reared under low stocking density were significantly heavier compared to those kept under high-density conditions (1068.0 g vs. 1024.3 g; *p* < 0.001). This difference significantly increased in the final weight (2527.9 g vs. 2466.1 g for low and high stocking densities, respectively; *p* = 0.019).

No significant differences were found between stocking density treatments in overall feed intake or mortality rate. However, birds reared under low stocking density consumed approximately 60 g less feed compared to those kept under high stocking density. Additionally, birds reared under low stocking density had a significantly better (lower) feed-to-gain ratio (1.39) compared with those reared at high density (1.45; *p* = 0.026). Regarding the interaction between probiotic level and stocking density, no significant effects were observed for any of the growth performance traits.

### 3.2. Carcass Yield and Lymphoid Organs

The effects of probiotic supplementation and stocking density on carcass characteristics and internal organ weights of broiler chickens are presented in Table 3. Neither probiotic supplementation nor stocking density had a significant effect on dressing percentage or carcass parts. All giblet organs showed a similar trend, except for the relative weight of the heart, where birds raised under low stocking density had a significantly (*p* = 0.038) higher heart percentage (0.46%) than those kept under high stocking density (0.42%). No significant differences were observed in the relative weights of the gizzard or liver due to either probiotic level or stocking density. Moreover, there was no significant probiotic × stocking density interaction for any of the measured carcass traits or internal organ weights.

### 3.3. Cell-Mediated Immunity

Table 4 presents the effects of probiotic supplementation levels and stocking density on the cell-mediated immune (CMI) response and lymphoid organ indices of broiler chickens. At 24 h post-challenge, probiotic supplementation had no significant effect on the CMI response, whereas stocking density showed a significant difference (*p* = 0.035). Broilers reared under low stocking density recorded a higher CMI value (58.3) compared to those under high stocking density (50.5). At 48 h, probiotic supplementation exhibited a significant quadratic effect (*p* = 0.017), while stocking density and their interaction were not significant. Birds supplemented with 0.2% probiotics had the highest CMI value (44.6), followed by those receiving 0.1% (41.0) and the control group (40.3). At 72 h, there was a tendency (*p* = 0.071) for higher CMI values in control birds; however, the difference was not significant. Stocking density again showed a significant effect (*p* = 0.017), with birds under low stocking density (28.1) displaying higher CMI values than those under high density (24.1). Regarding lymphoid organ indices, no significant effects of probiotic supplementation, stocking density, or their interaction were observed on the spleen, thymus, or bursa indices. All organ indices remained relatively similar across treatments, indicating that neither probiotic level nor stocking density markedly influenced lymphoid organ development.

$$\mathrm{Cell}\text{-}\mathrm{mediated}\;\mathrm{index}\;(\mathrm{CMI})=\frac{\mathrm{swelling}\mathrm{indunced}\mathrm{at}a\mathrm{tested}\mathrm{time}-\mathrm{ear}\mathrm{thickness}\mathrm{at}0\mathrm{time}}{\mathrm{ear}\mathrm{thickness}\mathrm{at}0\mathrm{time}}\times 100.$$

$$\mathrm{Lymphoid}\;\mathrm{organ}\;\mathrm{index}=\frac{\mathrm{lymphoid}\mathrm{organ}\;(g)}{\mathrm{live}\mathrm{body}\mathrm{weight}\;(g)}\times 100.$$

### 3.4. Blood Biochemical Parameters

Table 5 illustrates the effects of dietary probiotic supplementation and stocking density on the blood biochemical parameters of broiler chickens at 35 days of age. Probiotic supplementation had no significant effect on total protein, albumin, or globulin levels. However, stocking density significantly affected total protein (*p* = 0.011) and globulin concentrations (*p* = 0.007). Broilers reared under low stocking density showed higher globulin levels (2.09 g/dL) compared with those raised under high stocking density (1.72 g/dL), while the opposite trend was recorded for total protein levels. Broilers kept at high stocking density had significantly higher total protein levels (3.77 g/dL) than those at low stocking density (3.36 g/dL). Albumin levels were not significantly influenced by either probiotic level or stocking density. Regarding lipid profile, probiotic supplementation significantly influenced total lipid levels (*p* = 0.004, linear response), with the control group showing the highest value (1131.6 mg/dL), followed by the 0.1% and 0.2% probiotic groups, which exhibited significantly lower lipid levels (1014.2 and 1025.2 mg/dL, respectively). LDL concentration was also significantly affected by probiotic supplementation (*p* = 0.001, linear response), with the control group recording the highest LDL level (145.1 mg/dL), while the 0.1% and 2% probiotic groups showed markedly lower values (127.8 mg/dL and 134.0 mg/dL, respectively). Stocking density did not significantly influence total lipid or cholesterol levels, although birds under low stocking density exhibited numerically lower cholesterol and triglyceride concentrations. Triglyceride levels were significantly affected by stocking density (*p* = 0.035), with higher levels observed in birds under high stocking density (64.69 mg/dL) compared with those under low density (55.25 mg/dL). Additionally, broilers reared under low stocking density recorded significantly lower levels of LDL compared to those kept under high stocking density (130.4 vs. 140.9 mg/dL, respectively). A significant probiotic × stocking density interaction (*p* = 0.002) was also observed, suggesting that the combined effects of probiotic level and stocking density influenced triglyceride metabolism. HDL levels were not significantly affected by any of the treatments. Concerning antioxidant and oxidative stress markers, neither probiotic supplementation nor stocking density had significant effects on total antioxidant capacity (TAC) or malondialdehyde (MDA) levels. However, numerically higher TAC and lower MDA values were observed in birds reared under low stocking density, indicating a possible trend toward improved oxidative balance.

### 3.5. Ileal Histomorphology Measurements

**Figure 1:**
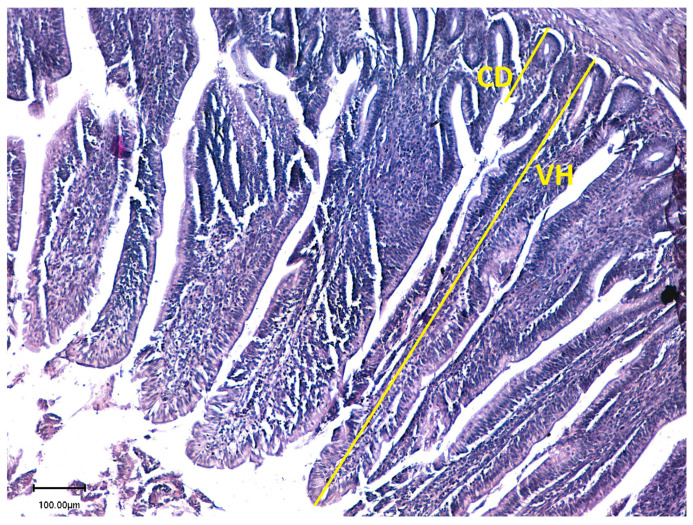
Villus height (VH) and crypt depth (CD) in histological section of un-supplemented group.

**Figure 2:**
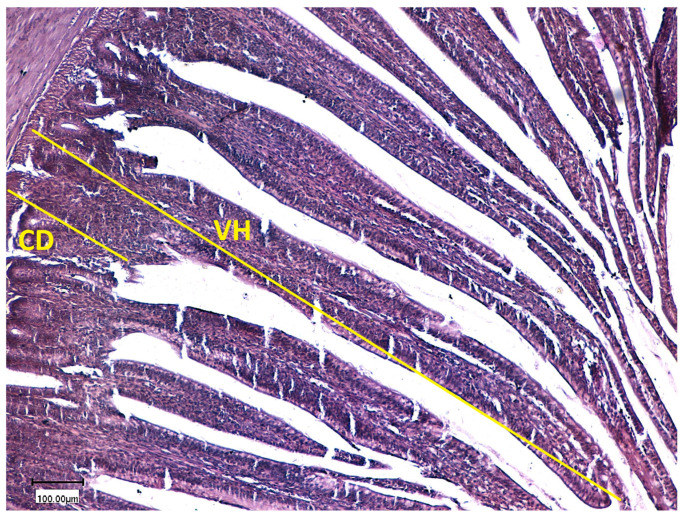
Villus height (VH) and crypt depth (CD) in histological section of broilers received low level of probiotics.

The effects of probiotic supplementation and stocking density on the ileal histomorphology of broiler chickens are summarized in Table 6. Probiotic supplementation had a highly significant linear effect (*p* < 0.001) on villus height. Birds receiving 0.1% probiotic showed the greatest villus height (1115.3 µm), followed by those given 0.2% probiotic (873.7 µm), while the control group recorded the shortest villi (680.5 µm). This indicates that probiotic inclusion markedly improved villus development. Stocking density also significantly influenced villus height (*p* = 0.012), with birds reared under low stocking density exhibiting taller villi (920.13 µm) compared with those kept at high density (880.4 µm). A significant interaction (*p* < 0.001) between probiotic level and stocking density indicated that the positive response in villus height was influenced by both probiotic supplementation and environmental conditions. Crypt depth was also significantly affected (*p* < 0.001, linear effect) by probiotic supplementation. The 0.1% probiotic group showed the deepest crypts (225.2 µm), followed by the 0.2% group (195.9 µm), while the control recorded the shallowest crypts (180.2 µm). Stocking density had a significant effect (*p* < 0.001) on crypt depth as well, with birds raised at high density showing deeper crypts (214.1 µm) than those at low density (188.7 µm). The interaction between probiotic level and stocking density was also significant (*p* = 0.007), suggesting that intestinal cell proliferation and turnover were influenced by both probiotic inclusion and crowding conditions. The villus-to-crypt ratio followed a similar trend, being significantly affected (*p* < 0.001) by both probiotic level and stocking density. Birds fed 0.1% and 0.2% probiotics exhibited higher ratios (5.03 and 4.80, respectively) compared with the control group (3.86). Low stocking density resulted in a greater ratio (5.06) than high density (4.14), and the interaction between probiotic supplementation and stocking density was highly significant (*p* < 0.001). Microscopic examination of ileal samples (Figure 1 and Figure 2) revealed that probiotic administration significantly increased villus height and decreased crypt depth in supplemented birds compared with the control group.

## 4. Discussion

### 4.1. Growth Performance and Mortality

Despite several reports on the effects of dietary probiotic supplementation on broiler growth performance, less attention has been given to the effect of the delivery route (via drinking water or feed). Probiotic supplementation through feed or water may potentially improve the production performance of broiler chickens, and water-soluble probiotics are considered more effective. Under heat stress, adding probiotics to drinking water can increase water intake in broiler chickens, which regulates body temperature, supports physiological functions and promotes overall metabolic efficiency. Probiotics, especially when added to drinking water, represent a promising feed additive to support gut microbial maturation and diversity, and may help reduce resistant bacteria in broiler chickens [21]. Many studies have shown that probiotic administration in poultry diets can significantly improve growth performance. Many studies have shown that probiotic administration in poultry diets can significantly improve growth performance [3,4,6]. Younas et al. [22] reported that both feed conversion ratio (FCR) and body weight gain were significantly improved in broilers fed a diet supplemented with two probiotic strains (*Enterococcus faecium* and *Weisella confusa*). Broilers receiving diets supplemented with either single or combined doses of *Lactobacillus plantarum* and *Bacillus subtilis* exhibited increased feed intake, higher weight gain, and improved FCR [23]. Similarly, the addition of *L. plantarum* B1 to the diet improved both weight gain and FCR in broilers [24]. In contrast, our results revealed that administering probiotics via drinking water did not significantly increase body weight throughout the experimental period. Consistent with the present findings, previous studies have demonstrated that probiotic supplementation did not significantly enhance body weight or growth performance in broilers [25,26].

High stocking density has economic benefits when maintained within an optimal range [14,27], as it reduces fixed production costs and increases meat yield per unit area, leading to greater overall profitability [28]. However, increasing stocking density beyond the optimal level results in poor air quality and higher ammonia emissions. Moreover, under high ambient temperatures, birds experience reduced heat dissipation from the body to the environment. Several researchers have reported reductions in body weight and poorer FCR in response to increasing stocking densities [27,29]. A clear pattern in our findings indicates that birds housed at lower stocking densities achieved higher body weights from week three onward, suggesting that the timing of intervention matters. This may be because younger birds are smaller and less affected by crowding, heat stress, or competition during early growth stages. Additionally, better feed conversion (lower F:G ratio) was observed in birds reared at lower densities compared with those at higher densities. However, exceeding the optimal density can negatively affect bird performance, welfare, and carcass quality. At excessively high densities, birds may experience stress due to limited space, poor air quality, and increased competition for feed and water, which can reduce feed efficiency and growth rate [30]. This agrees with previous studies reporting that high stocking density (HSD) in broiler production tends to reduce growth performance, increase stress, impair welfare, and decrease feed efficiency. In agreement with our findings, Cengiz et al. [29] reported that broilers reared at a high stocking density (20 birds/m^2^) had significantly lower feed intake and weight gain, along with a poorer FCR, compared with those reared at a lower stocking density (10 birds/m^2^). Similarly, Elkolaly et al. [31] stated that broilers raised under low stocking density (10 birds/m^2^) had significantly higher body weight and better FCR than those reared at high stocking density (16 birds/m^2^). The interaction between probiotic supplementation and stocking density on body weight is complex. No significant interaction was observed between stocking density and dietary probiotic supplementation for body weight gain, indicating that probiotics did not mitigate the negative effects of high stocking density [14,29,32]. Conversely, other studies have suggested that probiotic supplementation may alleviate the adverse impacts of high stocking density by reducing stress indicators and improving overall performance [33]. Likewise, Cengiz et al. [29] found that probiotics enhanced broiler performance during the starter phase at high stocking densities. Regarding the beneficial effects of probiotic inclusion on mortality, it was observed that probiotic supplementation reduced the mortality rate by approximately 2.4% and 2.9% under low and high stocking densities, respectively, compared with the control group. Consistent with our findings, a marked decrease in mortality was reported in broilers following probiotic administration [34]. This reduction suggests improved gut health, enhanced immune response, and better resistance to pathogenic microorganisms, leading to increased survival rates in broiler chickens. Reduced mortality directly enhances sustainability in poultry production by improving production efficiency and reducing resource wastage. Although the addition of probiotics evidently reduced mortality in broiler chicks, the difference was not statistically significant, which may be attributed to variation among replicate pens [35]. Similarly, the present results on mortality rate align with the findings of Fathi et al. [14] and Zhang et al. [6], who reported no significant differences in mortality rates among groups receiving dietary probiotics. In line with these observations, previous studies also indicated that stocking density had no significant effect on cumulative mortality rate [14,36].

### 4.2. Carcass Yield and Internal Organs

Most previous studies have concluded that the addition of probiotics to broiler diets has no significant effect on carcass yield or its components. In accordance with our results, the administration of multi-strain probiotics in broiler diets did not affect the relative weights of the carcass, breast, thigh, or giblet components compared with the unsupplemented group [3,26,37,38]. On the other hand, there is limited evidence suggesting that probiotics can improve carcass quality. Mehr et al. [39] reported that increasing the level of probiotic supplementation resulted in heavier body weight, a higher dressed carcass percentage, and an increased proportion of breast meat. The impact of probiotics on internal organ weights, however, remains unclear. No differences in the relative weights of the liver, spleen, gizzard, or heart were observed between broilers fed diets with or without probiotics [23,40]. Internal organ weights were generally unaffected by treatments, except for the heart, which was slightly heavier in birds reared under low stocking density. This may suggest that crowding stress at high densities suppresses cardiac development, possibly due to stress-induced physiological adjustments such as elevated corticosterone levels, which in turn may reduce organ growth [41].

### 4.3. Cellular Immunity

The impact of probiotics on immune function and health status is well-documented. Probiotic supplementation, particularly at the 0.2% level, improved the cell-mediated immune (CMI) response at 48 h, indicating a possible immunostimulatory effect. However, this effect was not consistent across all time points, suggesting that the influence of probiotics on cell-mediated immunity may depend on dosage, duration, or interactions with environmental factors such as stocking density. The positive effects of probiotic administration on the host immune response in broilers, through increased macrophage activity and enhanced phagocytic ability, are well established [42]. Enhanced immune function may lower the incidence and severity of infections, ultimately decreasing deaths among probiotic-treated broilers. Under high ambient temperatures, broilers fed dietary probiotics exhibited significantly higher serum IgM concentrations and improved cell-mediated immunity without compromising growth performance [14]. Similarly, the beneficial effects of probiotics on animal immunity have been demonstrated in other species; for instance, the inclusion of probiotics in rabbit diets increased cellular immune responses [43]. Probiotic-supplemented groups have also shown substantially elevated lymphocyte levels [22]. Dong et al. [44] observed that *Lactobacillus* strains enhanced T-helper 1 cytokine activity, while *Bifidobacterium* strains promoted anti-inflammatory immune responses. Thus, the use of dietary probiotics represents a promising strategy to maintain performance, intestinal health, and immunity, particularly under heat stress conditions [29,45].

According to the present results, stocking density had a more consistent influence on the cell-mediated immune (CMI) response than probiotic supplementation. Birds reared under low stocking density exhibited higher CMI responses at both 24 h and 72 h post-challenge, suggesting that reduced crowding may alleviate stress and enhance immune function. This trend is consistent with previous reports indicating that prolonged exposure to high stocking density suppresses immune responsiveness and elevates physiological stress markers [27,46]. The use of *Bacillus subtilis* and other lactic acid bacteria has been shown to enhance the immune response of broilers under stress. Guo et al. [47] reported that dietary supplementation with *Bacillus subtilis* (at 200 or 400 mg/kg) significantly increased serum IgM concentrations and improved cell-mediated immunity, even at elevated stocking densities (18 birds/m^2^), without compromising growth performance. Conversely, Mustafa et al. [48] found that stocking density did not influence humoral immunity in broilers. Similarly, Gholami et al. [49] reported that density had no effect on IgG, IgM, or total antibody levels against sheep red blood cells in broilers. In contrast, suppression of humoral immunity, reduced serum IgG, and significantly elevated heterophil-to-lymphocyte ratios have been observed in broilers raised at high densities [50]. Regarding lymphoid organ indices, no significant differences were detected due to either probiotic supplementation or stocking density. These results suggest that while functional immune responses (such as CMI) were affected, the overall development or size of the lymphoid organs remained relatively stable. Alkhalf et al. [51] and Naseem et al. [52] similarly reported that moderate probiotic supplementation did not significantly affect immune organ weights, despite improving immune response indicators. Likewise, the absence of significant effects of stocking density on lymphoid organs aligns with the findings of Simitzis et al. [53], who suggested that moderate increases in density may not necessarily impair immune organ development if management conditions are adequate. In contrast to our findings, Cengiz et al. [29] reported that the interaction between probiotic supplementation and stocking density (10 vs. 20 birds/m^2^) had no significant effects on key immune- or stress-related biomarkers, including nitric oxide concentration, heterophil-to-lymphocyte ratio, or lymphoid organ weights.

### 4.4. Ileal Histomorphology

**Figure 3:**
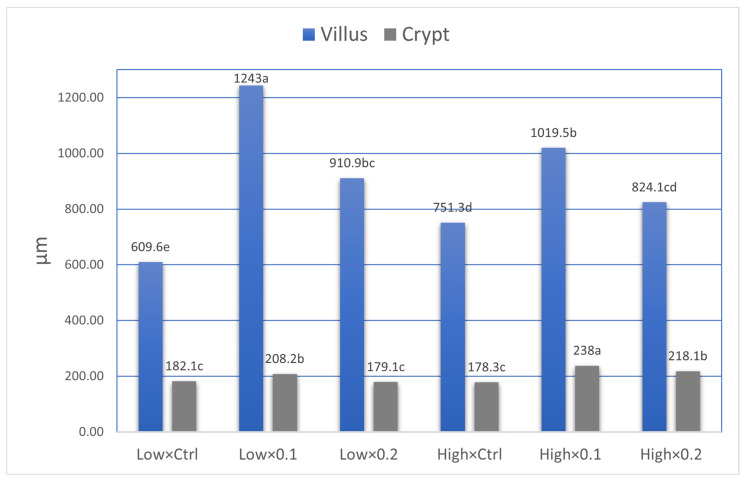
Effect of interaction between probiotic supplementation level (ctrl, 0.1and 0.2) and stocking density (Low and High) on villus height and crypt depth. ^a–e^ significance letters.

**Figure 4:**
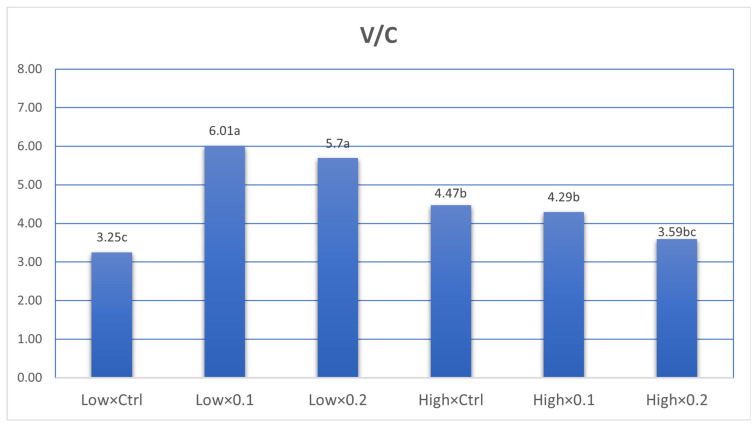
Effect of interaction between probiotic supplementation level (ctrl, 0.1and 0.2) and stocking density (Low and High) on villus: crypt ratio. ^a–c^ significance letters.

The present findings demonstrate that probiotic supplementation significantly enhanced ileal morphology, particularly at the 0.1% inclusion level. The observed increases in villus height and villus-to-crypt ratio indicate improved intestinal structure and absorptive surface area, suggesting better nutrient utilization and gut health. Longer villi provide a greater surface area for nutrient absorption, while moderate crypt depth reflects balanced epithelial cell proliferation and regeneration [19,54]. The improvement in villus morphology due to probiotic supplementation could be attributed to enhanced intestinal microbial balance. Probiotics help maintain beneficial gut microflora, suppress pathogenic bacteria, and produce short-chain fatty acids that nourish intestinal epithelial cells [51,55]. Hernandez-Coronado et al. [56] suggested that the improved small intestinal epithelial histomorphology observed in chickens fed probiotic-supplemented diets is associated with increased expression of tight junction proteins. Similarly, Pelicano et al. [57] reported that probiotics stimulate intestinal enzymatic activity and villus development, leading to more efficient nutrient digestion and absorption. Abdelqader et al. [58] also noted that broilers fed probiotics exhibited increased villus height and surface area, indicating a healthier intestinal environment. The deeper crypts observed in probiotic-supplemented birds may reflect greater enterocyte proliferation required to support the rapid turnover of intestinal cells [19]. However, the balanced villus-to-crypt ratio observed at 0.1% probiotic supplementation suggests that the rate of cell renewal is well matched with tissue maintenance, thereby optimizing gut function. Furthermore, there is significant interaction between the two factors. The birds received 0.1 probiotics and reared under low density recorded the highest height of villi compared with all sub-treatments (Figure 3). Stocking density also significantly affects intestinal morphology. Birds reared under low stocking density exhibited taller villi and higher villus-to-crypt ratios compared with those at high density, indicating that reduced crowding promotes better gut integrity and absorptive efficiency (Figure 1 and Figure 2). High stocking density is known to increase stress, alter intestinal microflora, and impair nutrient absorption [29,59]. According to Dozier et al. [27], higher bird density can modify intestinal structure due to physiological stress and reduced access to feed and space. The significant interaction between probiotic supplementation and stocking density suggests that probiotics may mitigate some of the adverse effects of crowding by improving gut morphology and maintaining microbial balance, as also reported by Dozier et al. [27] and Lan et al. [60]. The birds that received probiotics (both levels) under low stocking density recorded the highest V:C ratio among all sub-treatment groups (Figure 4). Administration of *Lactobacillus reuteri* has been shown to increase villus height, indicating that probiotics can enhance nutrient absorption and thereby improve growth performance and feed efficiency [34]. Furthermore, intestinal villus height and villus height-to-crypt depth (VH/CD) ratios were significantly increased in broilers fed diets supplemented with two probiotic strains (*Enterococcus faecium* and *Weisella confusa*) compared with the unsupplemented group [22]. Additionally, nutrient absorption improved as probiotics increased the absorptive surface area of the intestinal mucosa [61].

### 4.5. Blood Biochemical Analysis

Total protein, albumin, and globulin concentrations were not significantly influenced by probiotic supplementation. This finding agrees with the results of Abudabos et al. [62] and Yosi et al. [63], who reported that probiotics did not significantly alter serum protein fractions in broilers and ducks, respectively. Similarly, Alkhalf et al. [51] found that dietary probiotic supplementation (at 0.8 g and 1.0 g/kg) reduced serum cholesterol levels but did not significantly affect total protein, albumin, or total lipid concentrations. Broiler chickens given probiotics up to 21 days of age exhibited significantly lower plasma protein, total cholesterol, and HDL levels [64]. However, many parameters of the lipid profile and antioxidant status did not show significant differences for either factor. This may be due to the compensatory effects between probiotic inclusion and the negative impacts of crowding, thereby maintaining homeostasis in these parameters. Although total cholesterol levels were not significantly affected by either probiotic level or stocking density, a numerical decrease in cholesterol was observed with increasing probiotic levels. The lack of significant differences in the present study may be attributed to the relatively short experimental period or strain-specific variations in probiotic efficacy [54]. The improvement in LDL and total lipid values with probiotic addition aligns with previous findings showing that certain probiotic strains can reduce serum lipid concentrations and improve cardiovascular health markers in poultry. Similar results were reported by Kalavathy et al. [65] and Panda et al. [66], who found that *Lactobacillus*-based probiotics lowered serum cholesterol and LDL levels in broilers. This effect may be attributed to the bile salt hydrolase activity of probiotic bacteria, which deconjugates bile salts, thereby enhancing cholesterol excretion and reducing its intestinal absorption [67].

The positive effect on lipid metabolism was evident even independent of stocking density. These findings are consistent with reports that certain probiotic strains improve lipid metabolism and promote cardiovascular health in poultry [68,69,70]. Similarly, Pourakbari et al. [71] observed a linear reduction in triglyceride and cholesterol concentrations with increasing probiotic levels. These improvements in metabolic markers suggest that probiotics support nutrient assimilation and systemic homeostasis, likely through modulation of gut microbiota and enhancement of intestinal function.

**Figure 5:**
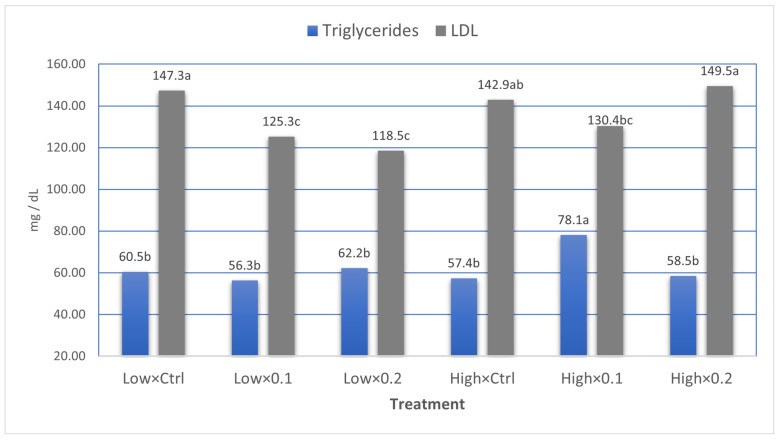
Effect of interaction between probiotic supplementation level (ctrl, 0.1and 0.2) and stocking density (Low and High) on triglycerides and LDL concentrations. ^a–c^ significance letters.

In terms of stocking density, a marked influence on protein metabolism was observed. Birds reared at low stocking density exhibited lower total protein and higher globulin concentrations, suggesting improved immune and metabolic status due to reduced stress and better nutrient utilization. High stocking density is often associated with environmental stress, which can suppress protein synthesis and impair immune function. The increased total protein and decreased globulin in birds raised under high stocking density may reflect an increase in other protein fractions (e.g., acute-phase proteins, albumin, or even muscle breakdown) rather than enhanced healthy protein synthesis. Alternatively, it may indicate dehydration or hemoconcentration under crowding stress, which can artificially elevate total blood protein. Total protein and albumin concentrations were not affected by probiotic supplementation [51,72]. High stocking density significantly increased triglyceride levels. Moreover, the interaction between probiotic level and stocking density was significant, suggesting that probiotic supplementation may modify the effects of density. The lowest level of LDL was found in birds receiving probiotics (both levels) under low stocking density (Figure 5). Similarly, the birds given 0.1 probiotics and kept under high stocking density recorded the highest level of triglycerides compared with all sub-treatments. It is well known that stress in birds, such as crowding, alters metabolism, including lipid metabolism, by increasing circulating free fatty acids and triglycerides [41,62]. Antioxidant indicators, including TAC and MDA, were not significantly influenced by treatments, although slight numerical improvements under low density suggest that reduced crowding may contribute to lower oxidative stress. The stable MDA levels across treatments indicate that lipid peroxidation remained within normal physiological limits, possibly due to adequate dietary antioxidants or moderate stress during experiment [73].

## 5. Conclusions

The findings of our study demonstrate that stocking density has a substantial impact on broiler growth performance, feed efficiency, immune response, and certain blood biochemical parameters, with low-density rearing being more favorable. Supplementation of drinking water with *Bacillus licheniformis* did not significantly affect growth performance or carcass traits. However, probiotic administration improved ileal histomorphology, including villus height, crypt depth, and the villus-to-crypt ratio. Probiotic supplementation in both levels (0.1–0.2%) enhanced lipid metabolism and reduced LDL concentration, while maintaining birds under low stocking density improved protein metabolism and immune-related globulin levels, thereby supporting better physiological status and overall health in broilers. The combination of probiotic supplementation, particularly at 0.1%, and low stocking density produced the most beneficial effects on intestinal health and immunity. These results highlight the importance of maintaining optimal stocking densities and using appropriate concentrations of probiotics as an effective strategy to support immune function, enhance flock health, and improve poultry production outcomes. Further studies are needed to explore the effects of multi-strain probiotics and different stocking densities under long-term heat stress on broiler performance.

## Figures and Tables

**Table 1:** The chemical analysis of basic diets and duration are described in the following table.

Ingredient, %	Starter 1–10 d	Grower 11–25 d	Finisher 26–35 d
Yellow corn	52	57	61.5
Soybean meal 44%	34	30	24.5
Wheat bran	4.5	4.5	4.1
Soy oil	4.0	4.5	6.0
Limestone	1.2	1.0	0.9
Dicalcium phosphate (DCP)	2.0	1.8	1.7
Salt (NaCl)	0.30	0.30	0.30
Vitamin–mineral premix ^1^	0.50	0.50	0.50
DL-Methionine	0.20	0.15	0.12
L-Lysine	0.30	0.25	0.40
Chemical composition			
Crude protein, %	22	21	20
Metabolizable energy, Kcal/Kg	2950	3050	3100
Moisture, %	12	12	12
Ether extract, %	6.8	7.3	8.9
Fiber, %	3.1	3.1	3
Calcium, %	0.9	0.8	0.8
Phosphor, %	0.7	0.7	0.7

^1^ Premix provided per kilogram of diet: 12,000 IU vitamin A, 3000 IU vitamin D_3_ (cholecalciferol), 30 IU vitamin E (*α*- tocopherol), 1.3 mg menadione, 2.2 mg thiamine, 8 mg riboflavin, 40 mg nicotinamide, 400 mg choline chloride, 10 mg calcium pantothenate, 4 mg pyridoxine, 0.04 mg biotin, 1 mg folic acid, 0.013 mg vitamin B12 (cobalamin), 80 mg Fe (ferrous sulphate), 8.0 mg Cu (copper sulphate), 110 mg Mn (manganese sulphate), 60 mg Zn (zinc oxide), 1.1 mg I (calcium iodate), 0.3 mg Se (sodium selenite).

**Table 2:** Growth performance of the broilers given different levels of probiotics under low and high stocking density.

Parameter	Probiotic Level (P)			Stocking Density (S)		SEM	*p*-Value			
	Control	0.1	0.2	Low	High		P		S	P × S
							Linear	Quad		
BW at 1d	40.7	40.5	40.4	40.5	40.6	0.14	0.548	0.550	0.799	0.698
3rd week body weight	1067.2	1031.7	1028.6	1068.0 ^a^	1024.3 ^b^	5.22	0.001	0.054	<0.001	0.054
5th week body weight	2488.5	2469.8	2512.0	2527.9 ^a^	2466.1 ^b^	12.69	0.494	0.180	0.019	0.701
BWG, g d1 to d21	1024.0	991.1	986.1	1022.0 ^a^	978.9 ^b^	9.14	0.105	0.218	0.013	0.342
BWG, g d21 to d35	1433.1	1424.3	1490.2	1453.8	1444.7	20.77	0.871	0.199	0.837	0.907
BWG, g d1 to d35	2457.1	2415.4	2476.3	2475.7	2423.5	11.47	0.428	0.380	0.229	0.813
FI, g d1 to d21	1257.7	1244.4	1265.9	1112.7 ^b^	1399.3 ^a^	28.0	0.557	0.449	<0.001	0.373
FI, g d21 to d35	2239.0	2187.9	2266.1	2344.0 ^a^	2118.1 ^b^	28.7	0.307	0.226	<0.001	0.592
Overall FI, g	3496.7	3432.3	3532.0	3456.6	3517.4	22.62	0.233	0.151	0.169	0.313
F:G, (g/g) d1 to d21	1.24 ^b^	1.26 ^ab^	1.29 ^a^	1.09 ^b^	1.43 ^a^	0.03	0.386	0.039	<0.001	0.401
F:G, (g/g) d21 to d35	1.57	1.54	1.52	1.62 ^a^	1.48 ^b^	0.02	0.609	0.474	0.004	0.972
Overall F:G, (g/g)	1.42	1.42	1.43	1.39 ^b^	1.45 ^a^	0.01	0.643	0.844	0.026	0.828
Mortality rate	5.3	2.9	2.4	4.3	3.0	0.79	-	-	-	-

FI = overall feed intake; F:G, feed to gain ratio. ^a,b^ Means with no common superscripts within each parameter represent significant difference (*p* < 0.05).

**Table 3:** Effect of probiotic supplementation and stocking density on carcass characteristics and internal organs weights of broiler chickens.

Trait ^1^	Probiotic Level (P)			Stocking Density (S)		SEM	*p*-Value			
	Control	0.1	0.2	Low	High		P		S	P × S
							Linear	Quad		
Dressing carcass	73.7	73.6	74.3	74.0	73.7	0.34	0.948	0.418	0.682	0.611
Whole leg	11.5	11.8	11.4	11.7	11.4	0.18	0.504	0.549	0.449	0.972
Thigh	6.9	7.1	7.0	7.1	6.9	0.15	0.567	0.843	0.637	0.988
Drumstick	4.6	4.7	4.5	4.7	4.5	0.07	0.742	0.447	0.107	0.975
Major pectoralis	11.3	10.9	10.8	11.0	11.1	0.16	0.374	0.462	0.722	0.839
Minor pectoralis	1.8	1.9	2.0	1.95	1.88	0.06	0.392	0.251	0.505	0.208
Heart	0.46	0.42	0.43	0.46 ^a^	0.42 ^b^	0.01	0.108	0.515	0.038	0.098
Gizzard	1.28	1.31	1.22	1.27	1.28	0.02	0.565	0.134	0.826	0.877
Liver	2.04	2.08	2.06	2.10	2.02	0.04	0.700	0.979	0.304	0.139

^a,b^ Means with no common superscripts within each factor are different (*p* < 0.05). ^1^ Carcass yield and internal organs are expressed as a relative figure of live body weight.

**Table 4:** Effect of supplemented probiotic level and stocking density on cell-mediated index and lymphoid organs of broiler chickens.

Parameter	Probiotic Level (P)			Stocking Density (S)		SEM	*p*-Value			
	Control	0.1	0.2	Low	High		P		S	P × S
							Linear	Quad		
CMI-24h	54.2	53.5	55.7	58.3 ^a^	50.5 ^b^	1.83	0.875	0.695	0.035	0.813
CMI-48h	40.3 ^b^	41.0 ^ab^	44.6 ^a^	42.9	40.9	0.79	0.726	0.017	0.216	0.764
CMI-72h	28.7	25.0	24.7	28.1 ^a^	24.1 ^b^	0.87	0.071	0.207	0.017	0.538
Spleen index	0.09	0.10	0.10	0.10	0.09	0.01	0.290	0.446	0.141	0.127
Thymus index	0.44	0.40	0.45	0.44	0.42	0.02	0.405	0.528	0.703	0.256
Bursa index	0.14	0.14	0.12	0.13	0.13	0.01	0.736	0.212	0.868	0.529

^a,b^ Means with no common superscripts within each factor are different (*p* < 0.05).

**Table 5:** Effect of supplemented probiotic Level and stocking density on blood biochemical analysis in broiler chickens.

Parameter	Probiotic Level (P)			Stocking Density (S)		SEM	*p*-Value			
	Control	0.1	0.2	Low	High		P		S	P × S
							Linear	Quad		
Total protein, g/dL	3.60	3.48	3.63	3.36 ^b^	3.77 ^a^	0.079	0.516	0.577	0.011	0.889
Albumin, g/dL	1.67	1.65	1.68	1.65	1.69	0.061	0.911	0.904	0.776	0.399
Globulin, g/dL	1.93	1.82	1.95	2.09 ^a^	1.72 ^b^	0.070	0.513	0.591	0.007	0.189
Cholesterol, mg/dL	168.41	160.00	155.48	156.73	165.66	2.88	0.197	0.146	0.108	0.310
Triglycerides, mg/dL	58.97	61.15	60.37	55.25 ^b^	64.69 ^a^	2.92	0.992	0.782	0.035	0.002
Total lipids (mg/dL)	1131.6 ^a^	1014.2 ^b^	1025.2 ^b^	1071.5	1046.2	16.9	0.004	0.134	0.463	0.899
LDL, mg/dL	145.1 ^a^	127.8 ^b^	134.0 ^b^	130.4 ^b^	140.9 ^a^	2.75	0.001	0.536	0.009	0.002
HDL, mg/dL	52.97	51.90	52.08	49.12	55.32	2.08	0.810	0.959	0.167	0.673
TAC (mM/L)	0.114	0.1988	0.122	0.166	0.121	0.03	0.229	0.549	0.393	0.222
MDA (nmol/mL)	0.9665	1.25	1.062	0.932	1.23	0.11	0.397	0.903	0.216	0.554

SEM = standard error of the mean, LDL = low-density lipoprotein, HDL = high-density lipoprotein, TAC = total antioxidant capacity, MDA = Malondialdehyde. ^a,b^ Means with no common superscripts within each factor are different (*p* < 0.05).

**Table 6:** Effect of supplemented probiotic Level and stocking density on ileal histomorphology in broiler chickens.

Ileal histomorphology	Probiotic Level (P)			Stocking Density (S)		SEM	*p*-Value			
	Control	0.1	0.2	Low	High		P		S	P × S
							Linear	Quad		
Villus height, µm	680.5 ^c^	1115.3 ^a^	873.7 ^b^	920.13 ^a^	880.4 ^b^	30.29	<0.001	0.308	0.012	<0.001
Crypt depth, µm	180.2 ^c^	225.2 ^a^	195.9 ^b^	188.7 ^b^	214.1 ^a^	4.02	<0.001	0.604	<0.001	<0.007
Villus: crypt ratio	3.86 ^b^	5.03 ^a^	4.80 ^a^	5.06 ^a^	4.14 ^b^	0.18	<0.001	0.613	<0.001	<0.001

^a–c^ Means with no common superscripts within each factor are different (*p* < 0.05).

## Data Availability

The data presented in this study are available within the manuscript.
